# Experimental and Theoretical Insights into the Intermolecular Interactions in Saturated Systems of Dapsone in Conventional and Deep Eutectic Solvents

**DOI:** 10.3390/molecules29081743

**Published:** 2024-04-11

**Authors:** Piotr Cysewski, Tomasz Jeliński, Maciej Przybyłek

**Affiliations:** Department of Physical Chemistry, Pharmacy Faculty, Collegium Medicum of Bydgoszcz, Nicolaus Copernicus University in Toruń, Kurpińskiego 5, 85-096 Bydgoszcz, Poland; tomasz.jelinski@cm.umk.pl (T.J.); m.przybylek@cm.umk.pl (M.P.)

**Keywords:** dapsone, solubility, deep eutectic solvents, green solvents, COSMO-RS, machine learning, intermolecular interactions, pattern recognition

## Abstract

Solubility is not only a crucial physicochemical property for laboratory practice but also provides valuable insight into the mechanism of saturated system organization, as a measure of the interplay between various intermolecular interactions. The importance of these data cannot be overstated, particularly when dealing with active pharmaceutical ingredients (APIs), such as dapsone. It is a commonly used anti-inflammatory and antimicrobial agent. However, its low solubility hampers its efficient applications. In this project, deep eutectic solvents (DESs) were used as solubilizing agents for dapsone as an alternative to traditional solvents. DESs were composed of choline chloride and one of six polyols. Additionally, water–DES mixtures were studied as a type of ternary solvents. The solubility of dapsone in these systems was determined spectrophotometrically. This study also analyzed the intermolecular interactions, not only in the studied eutectic systems, but also in a wide range of systems found in the literature, determined using the COSMO-RS framework. The intermolecular interactions were quantified as affinity values, which correspond to the Gibbs free energy of pair formation of dapsone molecules with constituents of regular solvents and choline chloride-based deep eutectic solvents. The patterns of solute–solute, solute–solvent, and solvent–solvent interactions that affect solubility were recognized using Orange data mining software (version 3.36.2). Finally, the computed affinity values were used to provide useful descriptors for machine learning purposes. The impact of intermolecular interactions on dapsone solubility in neat solvents, binary organic solvent mixtures, and deep eutectic solvents was analyzed and highlighted, underscoring the crucial role of dapsone self-association and providing valuable insights into complex solubility phenomena. Also the importance of solvent–solvent diversity was highlighted as a factor determining dapsone solubility. The Non-Linear Support Vector Regression (NuSVR) model, in conjunction with unique molecular descriptors, revealed exceptional predictive accuracy. Overall, this study underscores the potency of computed molecular characteristics and machine learning models in unraveling complex molecular interactions, thereby advancing our understanding of solubility phenomena within the scientific community.

## 1. Introduction

Dapsone (CAS Number: 80-08-0, IUPAC name: 4,4′-Diaminodiphenyl sulphone, abbreviated here as DAP) is a widely used [1] synthetic sulphone known for its anti-inflammatory and antimicrobial properties [2]. It is employed in both topical and systemic forms to treat various conditions, including leprosy, malaria, dermatitis herpetiformis, acne, and AIDS-concerned diseases [3,4,5,6,7,8]. Dapsone exerts its antibacterial effects by inhibiting the biosynthesis of folic acid through competitive inhibition of dihydropteroate synthetase, which ultimately hinders the production of nucleic acids essential for bacterial survival and multiplication [3,9]. The anti-inflammatory activity of dapsone can be attributed to the modulation of cytokine production [10,11]. The antioxidant potential of dapsone results from the reduced production of ROS and superoxide radicals by binding with NADPH oxidase [12,13]. Dapsone is metabolized in the liver via acetylation and hydroxylation and excreted primarily in urine [14,15]. Among its adverse effects [16,17], hematologic issues and peripheral neuropathy are considered the most important, with the dapsone hypersensitivity syndrome being identified as a life-threatening drug reaction [18,19]. Its low solubility in water and low permeability are the reasons for categorizing dapsone as a class II drug in the Biopharmaceutics Classification System (BCS) [20,21]. Because of the above features, the transdermal delivery route, instead of oral administration, is the preferred way of administering this drug [22,23]. Furthermore, transdermal delivery makes it possible to limit the adverse effects of dapsone, such as hemolyticanemia or nausea, which are the result of the production of metabolites in the liver [24]. These mentioned solubility limitations were the inspiration for various strategies aimed at resolving the poor aqueous solubility and low bioavailability of dapsone [25,26]. Additionally, the number of studies on the solubility of dapsone in various solvent systems [27,28,29,30] including neat solvents [28], as well as co-solvency systems [27].

Solubility, as a fundamental characteristic, holds significant importance in the pharmaceutical field and plays a crucial role in drug design and formulation [31,32]. The ability to dissolve in a particular solvent profoundly impacts a compound’s reactivity, stability, and bioavailability. Investigations into solubility, encompassing both equilibrium and kinetic methodologies, play a vital role in various pharmaceutical and chemical domains, including formulation of liquid dosage [33], improving bioavailability [34], facilitating crystallization [35], aiding pre-formulation [36], and enabling thermodynamic modeling [37]. It cannot be overstated that solvents are fundamentally important for the pharmaceutical industry, and it is estimated that they account for even 90% of all chemical forms [38]. Because of the amount of solvents used, it is crucial to focus not only on their effectiveness in dissolving active pharmaceutical ingredients but also on their environmental impact. This is why the paradigm of Green Chemistry [39,40] has become widely accepted also in the context of solvents [41,42,43,44]. Various properties can be attributed to these green solvents, with non-toxicity, non-flammability, and low environmental impact being the most important ones.

When considering the above properties, a particular group of designed solvents comes to mind, namely deep eutectic solvents (DESs). They are defined as mixtures of two or more components, i.e., a hydrogen bond acceptor (HBA) and a hydrogen bond donor (HBD), which can be solid or liquid, with a resulting melting point of the mixture lower than the ones of the individual components, which allows them to remain liquid at room temperature [45,46,47]. Deep eutectic solvents share many aspects with more traditional ionic liquids; however, they differ mainly by including non-ionic constituents in their structure [48]. Quite often DESs utilize such compounds as organic acids, alcohols, amino acids, sugars, or other plant-based primary metabolites [49,50]. The properties of DESs are in line with the general requirements for green solvents and include low volatility and flammability, sustainability and biodegradability, low cost, simplicity of preparation, and the potential for tuning them according to specific applications [47,51,52,53]. Recently, the ecotoxicity of DESs has been questioned and is therefore a crucial aspect of their application [54]. Several studies have evaluated their performance in this context, including the systems used in this work [55,56,57]. The desirable properties of DESs resulted in the widespread usage of deep eutectic solvents, and the pharmaceutical industry is, of course, one of the beneficiaries of this approach [58,59,60]. DESs have proven to have the ability to significantly increase the solubility and bioavailability of many APIs [61,62,63]. Also our research group has successfully demonstrated the usefulness of using DESs for the dissolution of many active substances, including sulfonamides [64], curcumin [65], caffeine [66], and recently edaravone [67]. Therefore, it seems natural to apply deep eutectic solvents also in the case of dapsone.

A detailed study on the solubility of dapsone in DESs requires not only experimental considerations, but also a theoretical analysis, which can help in gaining deeper insight into the nature of the considered systems, as well as in formulating predictive models [68,69]. In particular, an in-depth exploration of the solvent domain is a fruitful approach for screening solubilizers with the highest efficiency. Therefore, this work aims not only to provide new solubility data for dapsone, together with an analysis of intermolecular interactions for a large solubility data pool, but also applies a solvent screening approach based on machine learning.

## 2. Results and Discussion

### 2.1. Solubility of Dapsone in Deep Eutectic Solvents

**Figure 1:**
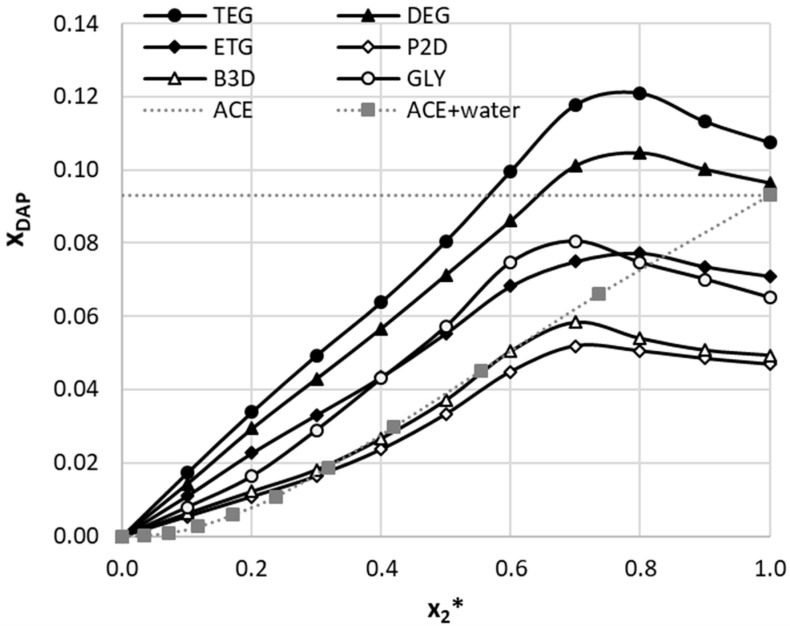
The solubility profiles of dapsone at room temperature (T = 25 °C) expressed as solvent composition-related mole fractions. The following notation was adopted: (1:2) choline chloride DESs with propane-1,2-diol (P2D), butane-1,3-diol (B3D) glycerol (GLY), ethylene glycol (ETG), diethylene glycol (DEG), triethylene glycol (TEG), and acetone (ACE)-water [27], where x_2_* stands for mole fractions of solute-free DES or ACE in aqueous mixtures.

The solubility of dapsone was studied here in several deep eutectic solvents comprising choline chloride (ChCl) and one of six non-toxic polyols, namely glycerol (GLY), ethylene glycol (ETG), diethylene glycol (DEG), triethylene glycol (TEG), 1,2-propanediol (P2D), and 1,3-butanediol (B3D). These systems were studied previously for other active pharmaceutical ingredients and proved to be very effective in enhancing their solubility [64,65,66]. Also, based on earlier experiences, the 1:2 molar ratio of DES constituents (i.e., a two-fold excess amount of the polyol) was used. Based on previously gained experience [64,65,66] that water can play a dual role as both co-solvent and antisolvent in certain concentration ranges, binary aqueous mixtures of DESs were additionally investigated. Hence, different molar proportions of the eutectics and water were prepared and used as composite solvents. Solubility was measured not only at 25 °C, but also at extended temperatures up to 40 °C. The obtained solubility values of dapsone in the studied systems were presented as solubility profiles in Figure 1 and the corresponding numerical values can be found in detail in Supplementary Tables S1.1.–S1.6.

When analyzing the obtained results, several interesting observations can be made. First of all, there is a following decreasing trend among the studied neat DESs in terms of dapsone dissolution effectiveness, namely TEG > DEG > ETG > GLY > B3D > P2D. This trend holds regardless of the temperature. At 298.15 K, the mole fraction solubility of DAP in the neat DES comprising choline chloride and triethylene glycol equals x_DAP_ = 0.1075. Of course, it is necessary to put the obtained results in the context of the solubility of DAP in traditional organic solvents [27,28]. It turns out that neat DESs outperform the majority of traditional organic solvents in terms of DAP dissolution efficiency. A prominent exception is acetone, which is reported to have a very high DAP solubility, equal x_DAP_ = 0.0882 [28] or x_DAP_ = 0.0931 [27]. This means that only two DESs, namely the ones utilizing TEG and DEG, are more efficient solubilizers than acetone. However, even with slightly lower dissolution abilities, they are still worth considering as real green alternatives to acetone. Also, quite expectedly, the increase in temperature of the samples results in elevated DAP solubility.

The studied DES systems differ slightly in their behavior when introduced as a part of an aqueous ternary solvent. It is worth commenting that the DES–water systems have a complex nature, reported for the first time in 2017 [70] in the case of a series of choline chloride/urea/water deep eutectic solvent mixtures. The systems are characterized by a nanostructure that is remarkably stable across a wide range of water contents. Water as amphiphilic compound actively modifies DES structure through the solvophobic capture of water into nanostructured domains around cholinium cations. Hence, despite the observed increase in the solubility trend with water dilution, it cannot be regarded as a typical co-solvent agent. Bearing this in mind, it is worth noting that the apparent co-solvency effects are observed for all DESs. This means that at a specific molar composition of the aqueous binary solvent, the solubility of dapsone is higher than for the pure DES. This is a very important factor since DESs are typically highly hygroscopic, which must be taken into account in laboratory practice. Fortunately, the moderate content of water even enhances the dissolution not only of dapsone but also many other APIs [64,65,66]. However, the solubility profiles are not identical among the studied DESs, as evidenced by Figure 1. The most important difference comes from the binary mixture composition, which results in the highest DAP solubility. In the case of eutectics comprising ethylene glycol, diethylene glycol, and triethylene glycol, the x*_DES_ = 0.8 composition is responsible for the highest solubility of dapsone. Meanwhile, for DES utilizing 1,2-propanediol, 1,3-butanediol, and glycerol, this composition is x*_DES_ = 0.7. The shape of the solubility profiles also differs slightly between the studied systems, although the solubility increase is rather similar and in the range about 10% greater when comparing the neat DES. It is also worth emphasizing that among the studied binary mixtures involving classical organic solvents [27] no co-solvency effect was observed, and only the mixtures of water and acetone result in DAP solubilities comparable to the ones found here. In fact, if the mole fraction of acetone in the aqueous mixture is smaller than 0.8, not only the DESs involving TEG and DEG perform better, but also those with ETG and GLY. Interestingly, even DESs comprising P2D and B3D initially have very similar solubilizing efficiency as acetone, in fact a better one in mixtures with high water content, and only after x*_ACE_ = 0.7 the organic solvent starts to perform better.

The differences in DAP solubility between the studied DES systems can be somewhat correlated with the structure of the considered polyols. The distinctly highest solubility was obtained for the systems comprising diethylene glycol and triethylene glycol, i.e., polyols with, respectively, one and two oxygen atoms replacing carbon atoms within their chains. This feature is not repeated within other polyols and may be the reason for their exceptional performance. Also, the hydroxyl groups of these two compounds are located at both ends of their chains. On the other hand, the two systems with the lowest solubilization efficiency of dapsone utilize 1,2-propanediol and 1,3-butanediol, which not only do not possess oxygen atoms within their chains, but one of their hydroxyl groups is not placed at the end of the aliphatic chain. In between, there are two polyols that differ significantly from one another, although they yield similar solubilities of DAP, namely glycerol and ethylene glycol. The aliphatic chain of glycerol does not contain oxygen atoms, and one of its hydroxyl groups is located on the middle carbon atom in the chain, which makes glycerol analogous to the two least-efficient polyols. However, glycerol has three hydroxyl groups in total, which may contribute to its increased solubility compared to P2D and B3D. Ethylene glycol, on the other hand, has two hydroxyl groups on both ends of its chain, similarly to the two best-performing compounds, although it does not have an oxygen atom within it, and thus results in a solubility lower than that of TEG and DEG.

### 2.2. Dapsone Solubility Dataset

**Figure 2:**
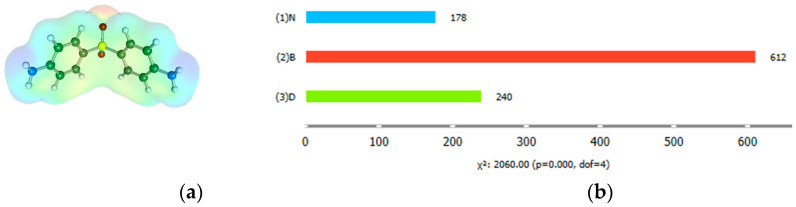
The structure of dapsone represented together with its distribution of electron density (**a**) and the dataset of dapsone solubility (**b**) expressed in terms of the logarithm of mole fractions for three sets of saturates systems, namely neat solvents, (1)N, binary solvents mixtures, (2)B, and ternary deep eutectic solvents, (3)D, including quaternary water-diluted ones.

Dapsone was frequently the subject of solubility measurements in various media, including water, alcohols, esters, and acetone. Indeed, the solubility in 26 neat solvents was reported, including methanol [27,28], ethanol [27,28], n-propanol [29], isopropanol [29], 1-butanol [28], isobutanol [28], 2-butanol [28], n-Pentanol [28], methyl acetate [28], ethyl acetate [27,28], isopropyl acetate [28], ethyl propionate [28], butyl acetate [28], acetone [27,28], 1,4-dioxane [29], 4-formylmorpholine (4FM) [30], DMSO [30], tetraethylene pentaamine [30], 1-methyl-2-pyrrolidone [30], diethylene glycol bis(3-aminopropyl) ether [30] and water [27]. In addition, there are available dapsone solubility values in binary mixtures with methanol [27], acetone [27], ethanol [27], n-propanol [29], isopropanol [29], 1,4-dioxane [29], and ethyl acetate-ethanol [27]. However, this comprehensive dataset only characterizes a small portion of the solvent hyperspace. Hence, the new measurements included in this work provide valuable extensions towards unexplored solvent regions. The supporting materials provide the details of the collection dataset and all experimental values used in this project, and Figure 2 presents a schematic overview of the dataset.

**Figure 3:**
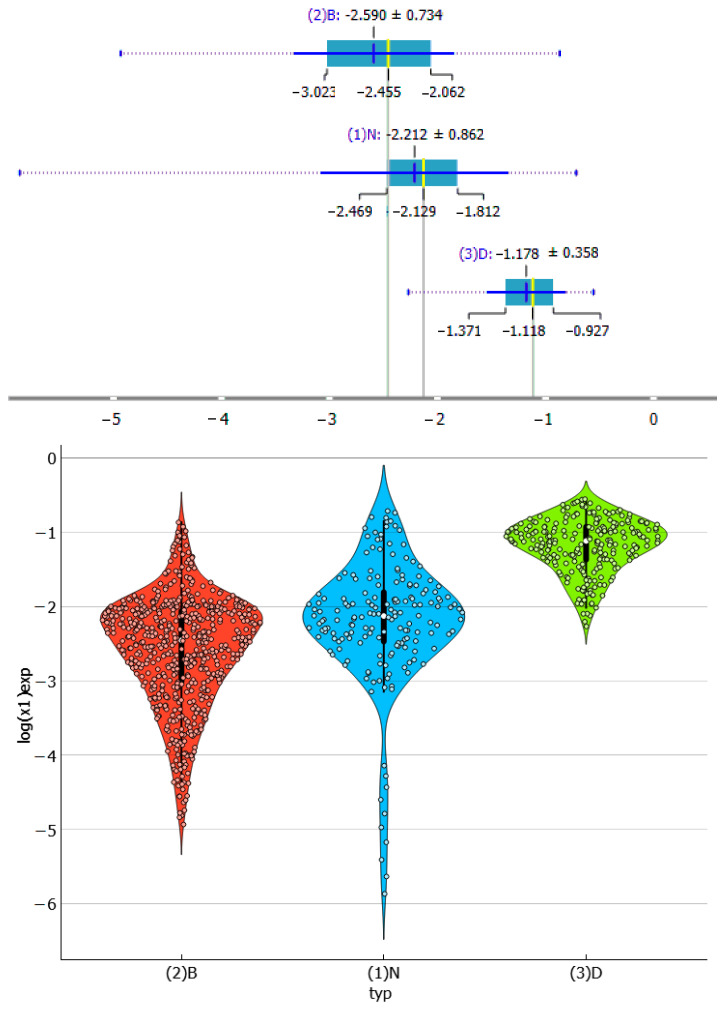
Distributions of the experimental values of dapsone solubility in three studied subsets presented in the form of box (**top**) and violin (**bottom**) plots.

Hence, the collection of dapsone solubilities is divided into three subsets: pure solvents, (1)N, binary solvent mixtures, (2)B, and binary deep eutectic solvents supplemented by ternary water-diluted eutectic systems, (3)D. The number of species in each subset, including their temperature and composition dependencies, is not uniformly distributed. The subset with the most experimental data points is the one collecting binary solvent mixtures. The other two categories are represented by a smaller but similar number of points. This may raise concerns about imbalance or misrepresentation, but from the perspective of this paper, it is not a serious limitation and does not affect the conclusions. The diversity of binary mixtures is rather limited and is also mostly covered in the neat solvent subset. Hence, binary solvent data do not introduce a bias. The solubility distributions are shown in Figure 3 as box and violin plots. As it might be anticipated, the three types of solvents exhibit significant differences. The box plot indicates that DES systems have the highest median solubility values, with log(x_D_) = −1.11, which is one logarithmic unit higher than the other two types of systems. It appears that the use of binary mixtures does not enhance the solubility of dapsone, as evidenced by the comparison of corresponding medians. This suggestion is also supported by the violin plot. The clearly detectable tail in the case of neat solvents corresponds to the lowest values represented by the dissolution of dapsone in pure water within the measured temperature range. Similarly, the tail in binary mixtures is associated with a high water content in the binary mixtures. Additionally, the box plot documents that the solubility data have the highest diversity for pure solvents. Indeed, some dissolution media, such as acetone or NMP, are as effective as many DES systems. This is also true for the low water content of these solvents when binary mixtures are used to dissolve dapsone. However, in general, the majority of DESs are superior to most other solvents. The only few exceptions are highly diluted DESs with a mole fraction of water equal to 0.9. It is worth mentioning that this kind of inferring with the identification of interesting cases can be performed straightforwardly using Orange data mining software using the attached file in supporting materials.

### 2.3. Distributions of Solute–Solute Affinity in Saturated Systems

The concentration- and temperature-dependent affinity values used to characterize the intermolecular interactions of dapsone in all saturated systems correspond to the values of Gibbs free energies of the pair formation reaction, as detailed in the methodology section. The dapsone dimerization in each of the investigated systems and under all experimental conditions, representing solute–solute interactions, is referred to as the AA type. The second reaction aims to characterize solute–solvent interactions by measuring the affinity for pairs formed between dapsone and one of the solvent molecules present in the system. In the case of a multicomponent solvent, all possible contacts are considered, and the affinity value is weighted according to the mole fraction of the solvent composition. This is referred to as the AB type contribution. The final group of molecular descriptors, BB, represents solvent–solvent interactions that are characterized by the formation of homo- or hetero-molecular pairs between solvent components. For instance, in the most complex cases of wet DESs, six combinations of interactions were considered, characterizing the dimerization of choline chloride, the second DES component, and water, in addition to three other distinct heteromolecular contacts. For binary mixtures, there are three different pairs, while the pure solvent only has one affinity contribution of this type.

For each system, the calculated Gibbs free energy values were also decomposed into two additional thermodynamic contributions that quantify the enthalpy and entropy of pair formation. These two data were often expressed in terms of the enthalpy-entropy compensation factor (EECF), which is defined as the ratio of the absolute enthalpy values to the sum of the absolute values of the enthalpy and entropic terms. This description enables the determination of the dominant affinity contribution under specific conditions. Values closer to unity indicate a higher enthalpic role, while values closer to zero indicate entropic dominance in the affinities of given systems. The contributions have been determined for all three types of interactions: solute–solute, solute–solvent, and solvent–solvent. This provides a detailed insight into the role of driving forces in each of the solubility datasets.

**Figure 4:**
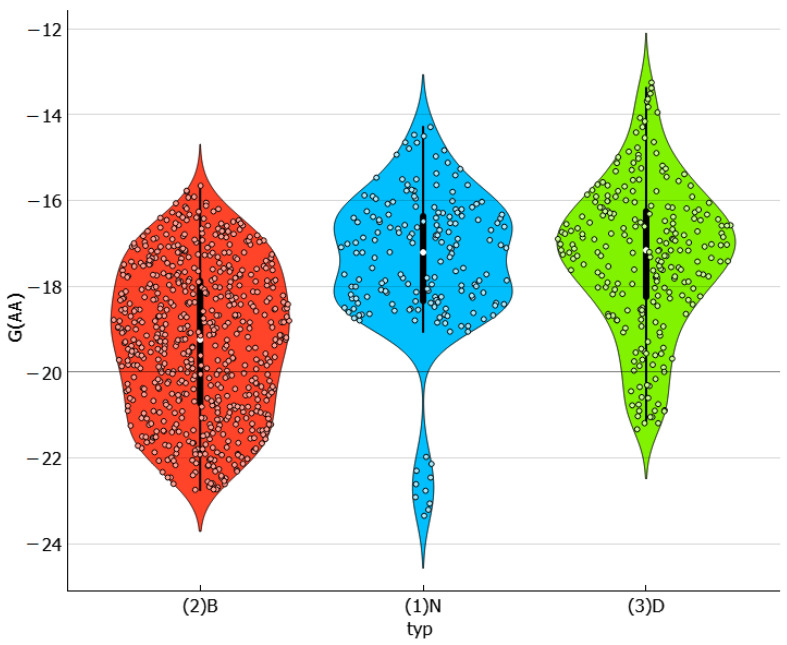
The characteristics of the dapsone self-association affinity expressed in the form of a violin plot. The values of the Gibbs free energies are expressed in kcal/mol.

**Figure 5:**
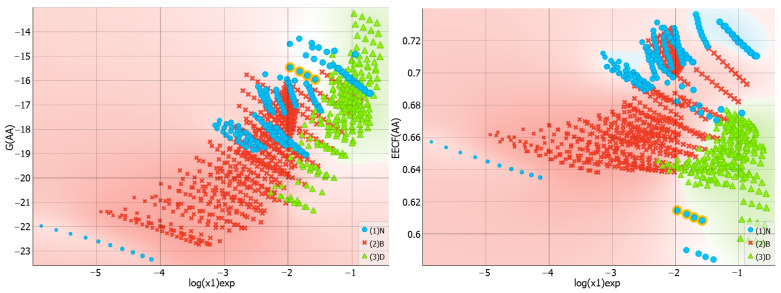
The distributions of self-association affinity values and corresponding EECF values as a function of solubility expressed in the form of logarithmic values of dapsone mole fraction. The values of the Gibbs free energies, G(AA), are expressed in kcal/mol.

Figure 4 presents the collected values of solute–solute interactions in the three studied systems in the form of a violin plot. It is noteworthy that dapsone dimerization is sensitive to the environment. As previously documented in one of our studies [30], the dapsone pair adopts a stacked conformation in all solutions. However, the stability is affected by the type of dissolution medium. It appears that this type of interaction is slightly more preferred in binary solvent systems. In addition to the similarity between the neat solvents and DESs is surprising, as shown by the almost identical density dot distributions on the violin plot. The violin plots illustrate this, indicating that, apart from the tail visible for neat solvents, the density points are almost equally distributed. The extreme values correspond to water solutions, where the self-attraction of dapsone is the strongest among all studied systems. This is expected since AA-type contacts are responsible for the aggregation of solute molecules, leading to precipitation. All these conclusions are additionally confirmed by the scatter plots presented on the left panel of Figure 5, showing a consistent trend of increasing solubility with a decreasing tendency toward dapsone self-association. The data characterizing the dissolution of dapsone in water are shown in the lower left corner, while the solubilities in water-free DESs with diethylene glycol are shown in the upper right corner. Furthermore, the right side of Figure 5 provides additional clarification of this observation. In all cases, the EECF(AA) is greater than half, indicating the dominance of energetic contributions in this type of interaction. This outcome is expected since self-association reduces the degrees of freedom of solute molecules, resulting in a decrease in the entropic contribution in favor of the enthalpic one.

### 2.4. Distributions of Solute–Solvent Affinity in Saturated Systems

**Figure 6:**
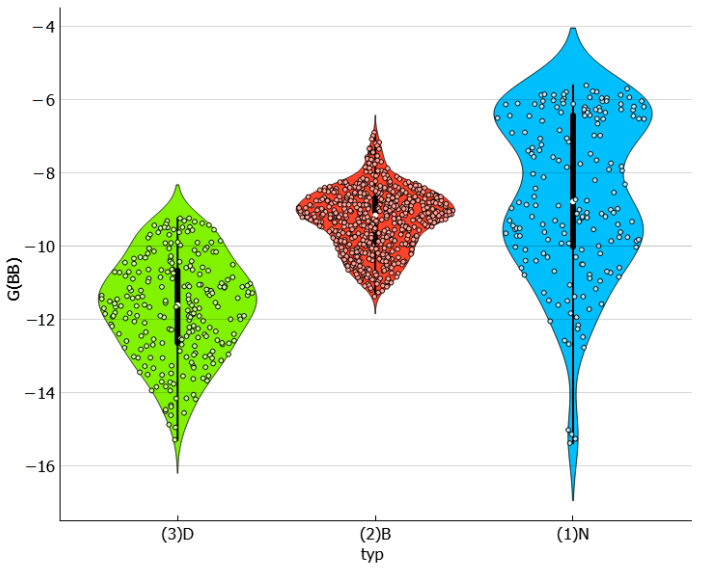
The characteristics of the solute–solvent affinity expressed in the form of a violin plot. The values of the Gibbs free energies are expressed in kcal/mol.

**Figure 7:**
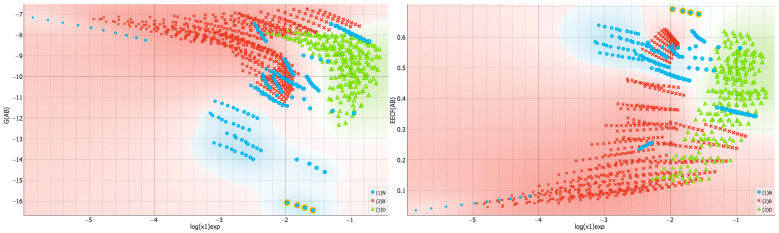
The distributions of solute–solvent affinity values and corresponding EECF values as a function of solubility expressed in the form of logarithmic values of dapsone mole fraction. The values of the Gibbs free energies, G(AB), are expressed in kcal/mol.

The importance of solute–solvent interactions cannot be overstated when it comes to stabilizing saturated systems. This is evident in the varying affinity values of dapsone toward different solvent molecules, as demonstrated in Figure 6. The greatest heterogeneity is observed in neat solvents, with the strongest attraction observed for TEPA differing by more than 10 kcal/mol compared to the weakest cases, which are water, acetone and butyl acetate. However, there is no direct relationship between solubility and the amount of these types of interactions. As shown in Figure 7, the distributions do not exhibit a definite trend, suggesting a more complex interplay of other factors on the effectiveness of dissolution. There are some interesting clues worth mentioning. For example, the majority of DESs, for which dapsone solubility is generally the highest, favor affinities in a quite narrow range between −12 and −8 kcal/mol. In contrast, the distribution is much more scattered for neat solvents due to the higher diversity of molecule types included in this set. For instance, TEPA exhibits the highest solute–solvent affinity among all neat solvents for dapsone. Conversely, water represents the opposite case.

### 2.5. Solvent–Solvent Interactions in Saturated Systems

**Figure 8:**
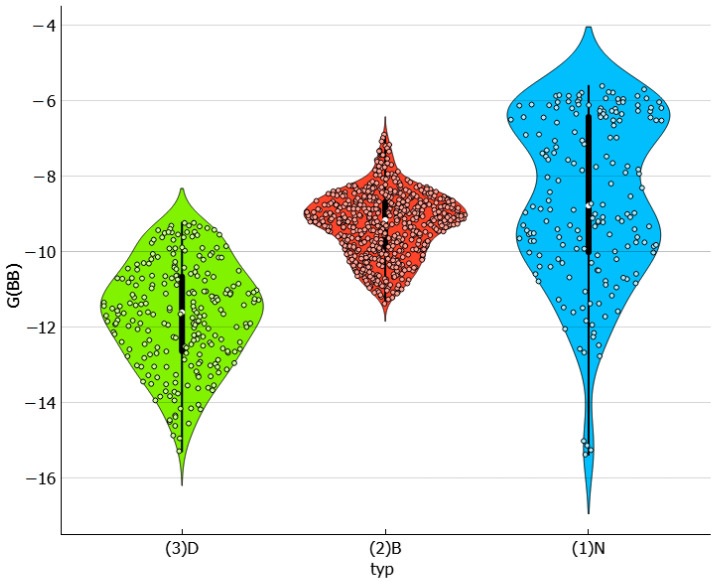
The characteristics of the solvent–solvent affinity expressed in the form of a violin plot. The values of the Gibbs free energies are expressed in kcal/mol.

The third type of interactions characterizes the mutual affinities of solvent molecules as the averaged values of solvent molecules self-association and hetero-molecular pairs formation. This type of interactions is supposed to be a potential indicator of readiness to solute dispersion by the environment and might be a crucial factor determining saturated system composition. Inspection of the overall distributions of solute–solute interactions presented in Figure 8 leads to a direct conclusion about the diversities of solvents. For example, the rather narrow range of types of molecules constituting the binary systems subset is clearly reflected in the very narrow span of affinities. To the contrary, the neat solvents subset exhibits much higher heterogeneity. What is a bit surprising is that DESs are quite diverse. In general, these solvents are characterized by the strongest internal stabilization forces. The visible tail on the violin plot for neat solvents corresponds to the strongest solvent–solvent interactions in the case of TEPA. All binary solvents are very similar if G(BB) values are taken into account.

**Figure 9:**
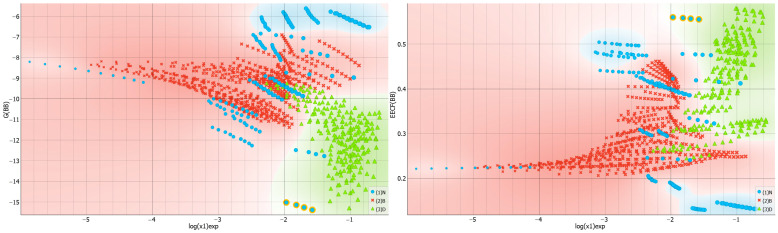
The distributions of solvent–solvent affinity and corresponding EECF values as a function of solubility, expressed in logarithmic values of dapsone mole fraction. The Gibbs free energies, G(BB), are expressed in kcal/mol.

**Figure 10:**
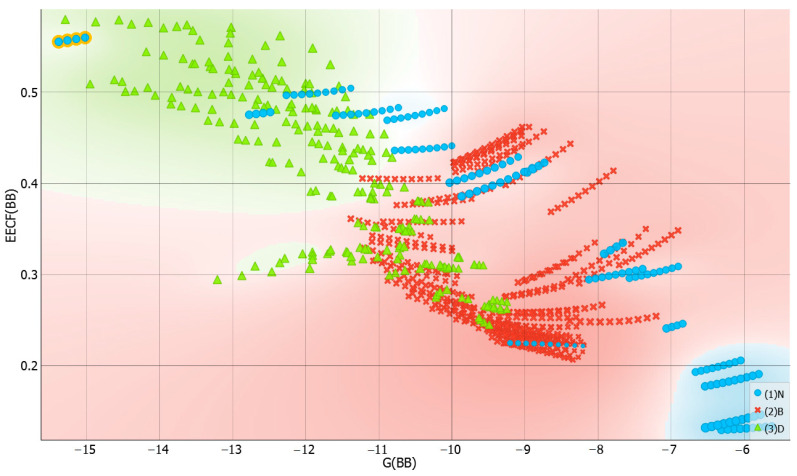
The correlation between solvent–solvent interactions and corresponding EECT values.

It is interesting to inspect if there is any detectable trend in solvent–solvent interactions and dapsone solubility. This is visualized in Figure 9, which collects both the distributions of affinity and EECF(BB) values for this type of interactions. First of all, it is interesting to notice that systems with high dapsone solubility are in general characterized by quite diverse solute–solute affinities, and only TEPA and diethylene glycol bis(3-aminopropyl) ether (B3P) are similar in this respect. However, there are systems like acetone and the majority of esters, which are also very effective solvents for dapsone, but their intermolecular interactions determining internal structure are quite low. This can be understood in light of the mechanism of dapsone dissolution, already discussed in our earlier works [30,71]. It was argued that dapsone can interact via either hydrogen bonding motives or, due to the quite large hydrophobic part of the molecule, via non-specific interactions of dispersion nature. Hence, such bivalent ability allows for a variety of mechanisms of dissolution, even with distinct solvents. In the right part of Figure 9, the EECF distribution is presented for solvent–solvent interactions. It is interesting to note that both presented trends are of the opposite character. Hence, inspection of the direct relationships between G(BB) and EECT(BB) seems to be worth presentation. Indeed, this is documented in Figure 10 by the plot correlating the values of solvent–solvent affinities with the enthalpy-entropy compensation factor, EECF. It is interesting to notice that there is a fairly linear trend between these two values, and the increase in G(BB), characterizing the decreasing affinity, is associated with the decrease in EECF(BB). This suggests that such solvents, which are characterized by low self-affinities, in general are more disordered, as inferred from the higher contribution from the dominance of entropic contributions. Indeed, the left-bottom corner is occupied with light esters and acetone, i.e., non-protic systems. In such cases, the values of EECF reach 20% at most. On the contrary, for protic components, such as TEPA or many DESs, the enthalpic contribution dominates, exceeding 50%. This emphasizes the fact that the enthalpy factor is crucial for enhancing the solvent–solvent interactions.

### 2.6. Machine Learning Models

**Figure 11:**
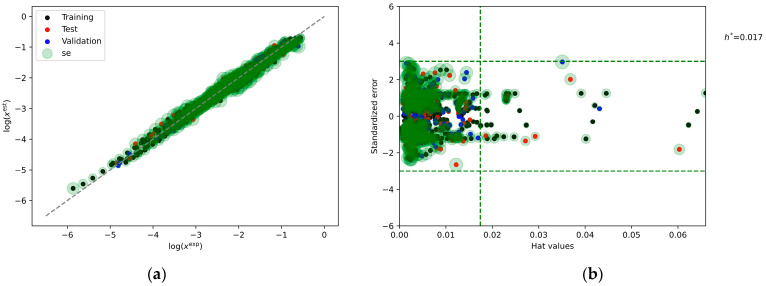
(**a**) The representation of the accuracy of the developed NuSV regression model characterizing the relationship between computed and measured logarithmic values of dapsone mole fraction in saturated conditions. The values of the optimized model parameters are the following C = 9.995195013432355, degree = 2, gamma = 0.6489637022275867, and nu = 0.13135730736919388. The importance of the descriptors was found to be as follows: G(AA) = 1.33, G(AB) = 0.46, G(BB) = 0.69, EECT(AA) = 0.42, EECT(AB) = 0.49, and EECT(BB) = 0.82. (**b**) The applicability domain analysis as a qualitative diagnostic metric is provided.

The foregoing discussion underscored the significance of molecular affinities and highlighted the heuristic value inherent in the computed characteristics, offering valuable insights into the molecular origins of observed solubility. The utility of these computed values can be further enhanced by showcasing their predictive potential through the development of machine learning models. In this study, among a plethora of potentially useful regressors, the NuSVR (Non-Linear Support Vector Regression) was judiciously chosen as an exemplary non-linear model for predicting the solubility of dapsone across various solvent systems. The prediction of the trained model is presented in Figure 11, revealing a commendable level of accuracy. Notably, the MSEs are strikingly small, with values of 0.014, 0.018, and 0.019 for the training, test, and validation subsets, respectively. The model’s robustness is further affirmed through the incorporation of applicability domain analysis, as depicted in Figure 11b, where the standardized error is plotted as a function of hat values. Although hat values are commonly used in the context of linear models they can still provide a useful characterization of the influence and leverage of data points also for non-linear models. Indeed, data points with high hat values have a larger influence on the learned decision function. The hat values are not used for inference purposes, but as a diagnostic metric to flag observations that may be outside the applicability domain of the model. This analysis indicates that all predicted values across the three subsets fall within an acceptable range, delimited by three times the standard deviation and represented by dashed lines. Additionally, it is noteworthy that dapsone self-association emerges as the most influential descriptor, followed closely by the EECT(BB), the significance of which in relation to solubility has been previously acknowledged.

## 3. Materials and Methods

### 3.1. Materials

Dapsone (DAP, CAS Number: 80-08-0) was acquired from Sigma Aldrich (Saint Louis, MO, USA) with a purity of at least 99%. The constituents of DESs were also obtained from Sigma Aldrich with a purity of no less than 99%. These constituents include choline chloride (ChCl, CAS Number: 67-48-1), glycerol (GLY, CAS Number: 56-81-5), ethylene glycol (ETG, CAS Number: 107-21-1), diethylene glycol (DEG, CAS Number: 111-46-6), triethylene glycol (TEG, CAS Number: 112-27-6), 1,2-propanediol (P2D, CAS Number: 57-55-6), and 1,3-butanediol (B3D, CAS Number: 107-88-0). Methanol (CAS Number: 67-56-1) used as a solvent throughout this study was supplied by Avantor Performance Materials (Gliwice, Poland) and had a purity of at least 99%. Prior to use, choline chloride was dried, while all the other compounds were used without any initial procedures.

### 3.2. Methods

#### 3.2.1. Preparation of the Samples and Solubility Measurements

In order to determine the solubility of dapsone in the studied DESs, the initial step involved the preparation of a calibration curve. For this purpose, a stock solution of DAP was prepared in methanol and subsequently diluted in 10 mL volumetric flasks. The concentration of solutions used for the preparation of the calibration curve ranged from 0.007 mg/mL to 0.017 mg/mL. These solutions were measured spectrophotometrically utilizing an A360 spectrophotometer from AOE Instruments (Shanghai, China). The wavelength corresponding to the maximum absorbance value was found to be 295 nm. The final curve was the result of averaging three separate curves. The linear regression equation was found to be A = 113.8664∙C + 0.0015 (A—absorbance, C—concentration expressed in mg/mL). The determination coefficient indicated a high degree of linearity, with R^2^ = 0.999.

In the investigation of various deep eutectic solvents, choline chloride consistently served as one of the constituents. The second component among the six mentioned compounds, namely glycerol (GLY), ethylene glycol (ETG), diethylene glycol (DEG), triethylene glycol (TEG), 1,2-propanediol (P2D), and 1,3-butanediol (B3D) varied. To formulate a DES, choline chloride was mixed with the second compound in different molar ratios within sealed test tubes. These tubes were then placed in a water bath at 363.15 K until a homogeneous solution formed. The resulting DESs were used either in their pure form or combined with water to create binary systems with varying water proportions. To obtain saturated solutions of dapsone (DAP) in the studied systems, excess amounts of DAP were added to the test tubes containing either a pure DES or binary mixtures with water.

The well-established shake-flask method was used in this study to determine the solubility of dapsone in the considered systems. This particular method was used numerous times by our group [72,73,74], and its reliability was consistently confirmed by comparison with literature data.

The prepared samples were incubated for 24 h at four different temperatures, namely 298.15 K, 303.15 K, 308.15 K, and 313.15 K, in an Orbital Shaker Incubator ES-20/60 from Biosan (Riga, Latvia). The temperature was precisely maintained at 0.1 K, with a variation of ±0.5 K observed over the 24 h cycle. During the mixing process, all samples were agitated at a speed of 60 rev/min. Due to the higher viscosity and density of the deep eutectic solvent systems, centrifugation was necessary. The samples underwent centrifugation at 1000 rev/min for 5 min using the EBA 20 centrifuge from Hettich (Tuttlingen, Germany). This process ensured that any undissolved precipitate settled at the bottom of the test tubes. Afterward, the samples were filtered using a syringe equipped with a PTFE syringe filter featuring a pore size of 0.22 µm. To avoid precipitation, the test tubes, syringes, pipette tips, and filters were initially heated to match the temperature of the handled sample.

Finally, small volumes of the filtered solution were transferred to test tubes containing methanol, and the diluted samples were measured using a spectrophotometer. Additionally, to determine the mole fractions of dapsone, the density of the samples was measured by weighing a fixed 1 mL volume of the solution in 10 mL volumetric flasks. Throughout this study, an Eppendorf (Hamburg, Germany) Reference 2 pipette was used with a systematic error of 0.6 µL. The RADWAG (Radom, Poland) AS 110 R2.PLUS analytical balance with a precision of 0.1 mg was also utilized.

To determine the solubility of dapsone in the studied systems, the samples prepared as per the previously mentioned procedure underwent a spectrophotometric analysis using the same spectrophotometer as was the case for the calibration curves. The spectral data were recorded within the wavelength range of 190 nm to 500 nm, with a resolution of 1 nm. Initially, methanol was used for spectrophotometer calibration, and it was also utilized to dilute the measured samples. Dilution was necessary to ensure that the absorbance values remained within the linear range. Specifically, the absorbance values at 295 nm were considered, and based on the previously prepared calibration curve, the concentration of dapsone in the samples was determined, along with its mole fractions. These values were obtained by averaging the results from three separate measurements.

#### 3.2.2. Intermolecular Interaction Quantification

This study utilizes the widely used COSMO-RS framework [75,76] and leverages the established methodology employed in our recent works [67,71,77,78,79] to quantify intermolecular interactions within the investigated saturated systems. While the core computational framework remains analogous, this work incorporates solvent–solvent affinities alongside the previously considered solute–solute and solute–solvent interactions. These affinities are expressed as the values of the Gibbs free energies (ΔG) for the reaction X + Y → XY, where X and Y represent either dapsone or any solvent component. Notably, for dapsone dimerization, both reactants (X and Y) are defined as the solute molecule (DAP). Conversely, solute–solvent affinities reflect the formation of heterodimeric pairs, where X denotes dapsone and Y represents a molecule from the neat solvent, a binary organic solvent mixture, a binary deep eutectic solvent formulation, or a ternary aqueous DES mixture. Affinity values for every pair in every system and under every experimental condition were determined by computing the concentration-dependent Gibbs free energies using COSMOtherm software (Version 24.0.0) [80]. It is important to note that the molecular descriptors characterizing the intermolecular interactions were derived in such a way that, irrespectively of the number of components in the solvents, only three values are collected for the accounting of solute–solute, solute–solvent and solvent–solvent affinities. These molecular descriptors were determined by weighting the given pair interaction by the mole fraction of the solvent mole fraction in solute-free mixtures. For example, the descriptor characterizing the overall solute solvent interactions (AB) in binary mixtures was obtained from individual contributions AB = x_2_*·AB_1_ + (1 − x_2_*)·AB2, where x_2_* and subscripts represent the numbering of the solvents. In such a manner, the smooth functions of the solute–solvent and solvent–solvent interactions were obtained for the whole range of solvent compositions. Additionally, this guaranteed a smooth trend when binary mixtures were replaced with neat solvents.

#### 3.2.3. Conformational Analysis

To ensure that systems are characterized by the most representative structures, an extensive conformational analysis preceded the calculation of affinity values. This involved a search for the lowest-energy conformations of all 28 monomeric components using the COSMOconf package (Version 24.0.0) [81]. Each molecule was represented by a maximum of ten low-energy conformations, adhering to the standard protocol employed in COSMO-RS theory within COSMOtherm. Notably, independent conformational searches were conducted for both the gas and condensed phases. The latter is crucial to account for the influence of the surrounding environment within the conductor-like screening model. Since the latest COSMOtherm database lacked some of the required structures, particularly for pairs configurations, all monomer conformations were newly generated to maintain consistency. The final step yielded “cosmo” and “energy” files compatible with the BP_TZVPD_FINE_21.ctd parameter set, representing the highest level of detail available in COSMOtherm, corresponding to RI-BP/TZVP//TZVPD-FINE level computations.

#### 3.2.4. Conformational Search for Pairs Structures

Identification of structurally diverse pairs posed a more significant computational challenge. This involved generating conformations for various combinations: dapsone dimers, dapsone-solvent pairs (27 sets), solvent dimers (27 sets), and most demandingly, all possible combinations of 351 solvent hetero-pairs. To achieve this, the COSMOtherm software employed the “CONTACT = {1 2} ssc_probability ssc_weak ssc_ang = 15.0” command. This command prioritizes the generation of the most probable pairs based on contact probabilities, considering both hydrogen bonding and weak interactions. Consequently, this step typically results in a substantial number of initial structures requiring further optimization. Geometry optimization was performed at the RI-DFT BP86 (B88-VWN-P86) level to refine these initial structures. Subsequently, data reduction was implemented to eliminate redundant and high-energy geometries. Two criteria were used for this selection: root-mean-square deviation (RMSD) and relative energy compared to the most stable conformer. Ultimately, only unique contacts within a 2.5 kcal/mol energy window relative to the most stable structure were retained for each complex, ensuring a representative pool of conformers with diverse structures and energies. This comprehensive procedure was applied to generate all types of pairs utilized in this study, resulting in a final set of structures that effectively captures the molecular diversity relevant to this project. The final “cosmo” and “energy” files were generated on the same level of theory and used for affinity computations using COSMOtherm facilities.

#### 3.2.5. Pattern Identification with Orange Data-Mining

Orange data mining software [82] was employed to identify the most significant patterns within the intermolecular interaction dataset. This software facilitated the exploration and analysis of the data, enabling the extraction of key relationships between the various parameters. The specific details of the data, feature selection, and chosen pattern mining used within Orange are elaborated in the supporting material, along with the full dataset and code for further free exploration. Refer to Section S2 in the Supplementary Materials for details.

#### 3.2.6. Machine Learning Modelling

The predictive model for solubility was developed utilizing an in-house Python code (version 3.10, https://www.python.org/) specifically crafted for hyperparameter tuning across a variety of regression models. For the purpose of this project, the Nu-Support Vector Regression (NuSVR) was selected due to its well-recognized predictive ability. The performance of this regression model relies on the appropriate tuning of the following parameters:

- Regularization parameter (C) that controls the trade-off between achieving a smooth decision boundary and fitting the training data;
- Degree for polynomial kernel;
- Gamma defining the influence of a single training example and affects the shape of the decision boundary;
- nu parameter, which is specific to NuSVR and represents an upper bound on the fraction of margin errors and a lower bound on the fraction of support vectors. It is a crucial parameter that influences the model’s flexibility and generalization.

The exploration of the hyperparameter space to identify optimal values was facilitated through the Optuna study (version 3.2, https://optuna.org/), a freely available Python package for hyperparameter optimization. The optimization process involved 5000 minimization trials utilizing the tree-structured Parzen estimator (TPE) as the search algorithm sampler. TPE, recognized for its computational efficiency, utilizes a probability density function to model the relationship between hyperparameters and performance metrics. In assessing the performance of the NuSV model, a custom score function was implemented, amalgamating multiple metrics to address both accuracy and generalizability, as defined in previous work [67,77,78,79], with detailed mathematical insights provided therein. The scoring function’s distinctive feature lies in the incorporation of penalties derived from learning curve analysis (LCA) within the scikit-learn 1.2.2 library during parameter tuning. Given the computational expense of LCA, two-point computations were executed, involving 50% and 100% of the total data. The values integrated into the custom loss function correspond to the average Mean Absolute Error (MAE) values obtained at the largest training set size. Consequently, this custom loss function combines both components, offering insights into the model’s accuracy and its ability to generalize to novel, unseen data. The dataset was partitioned into training (70%), testing (15% and validation (15%) for comprehensive model evaluation.

## 4. Conclusions

In this study, the solvents’ hyperspace has been expanded by incorporating new dapsone solubility data obtained in choline chloride-based deep eutectic solvents (DESs) with six polyols. This further augmented the comprehensive dataset, encompassing 27 neat solvents and 6 binary mixtures, with newly measured deep eutectic solvents. Our dataset not only captures solubility dependencies on solvent conditions but also considers the influence of temperature variations. To elucidate the molecular origins of observed solubility profiles, sets of molecular descriptors were computed characterizing affinities among system components. Utilizing these descriptors for pattern recognition unveiled intriguing relationships between solubility and affinity contributions. Notably, this study highlights the pivotal role of dapsone self-association as a key determinant of solubility, a finding reinforced by its highest importance in the developed Non-Linear Support Vector Regression model. Solute–solute affinities, particularly the enthalpy-entropy compensation factor, were identified as crucial determinants of solubility profiles.

This investigation underscores the essential role of molecular affinities in comprehending solubility, leveraging computed characteristics to delve into the molecular origins of observed patterns. The predictive potential of these computed values is demonstrated through the implementation of the NuSVR model for dapsone solubility prediction across diverse solvent systems. The NuSVR model exhibits outstanding accuracy, yielding minimal Mean Squared Errors (MSEs) of 0.014, 0.018, and 0.019 for the training, test, and validation subsets, respectively. Rigorous applicability domain analysis further validates the model’s robustness, confirming all predicted values within an acceptable range. It is noteworthy that the set of molecular descriptors utilized in this study is unique and not previously explored in the scientific literature. The insightful results, obtained both at a heuristic level and as suitable representations for machine learning model development, serve as a promising foundation for future advancements and extensions. This study underscores the potency of computed molecular characteristics and machine learning models in unraveling complex molecular interactions, thereby advancing our understanding of solubility phenomena within the scientific community.

## Data Availability

All data supporting the reported results are available on request from the corresponding author.

## Supplementary Materials

The following supporting information can be downloaded at: https://www.mdpi.com/article/10.3390/molecules29081743/s1, Section S1. Collection of experimental data of dapsone solubility. Table S1.1. Mole fraction solubility of dapsone (x_DAP_, ∙10^4^) in mixtures of water and DES containing choline chloride and 1,3-butanediol (B3D) at various temperatures. In the first column, x*_B3D_ denotes the mole fraction of DES in solute-free mixtures with water. Standard deviation values are given in parentheses. Table S1.2. Mole fraction solubility of dapsone (x_DAP_, ∙10^4^) in mixtures of water and DES containing choline chloride and 1,2-propanediol (P2D) at various temperatures. In the first column, x*_P2D_ denotes the mole fraction of DES in solute-free mixtures with water. Standard deviation values are given in parentheses. Table S1.3. Mole fraction solubility of dapsone (x_DAP_, ∙10^4^) in mixtures of water and DES containing choline chloride and glycerol (GLY) at various temperatures. In the first column, x*_GLY_ denotes the mole fraction of DES in solute-free mixtures with water. Standard deviation values are given in parentheses. Table S1.4. Mole fraction solubility of dapsone (x_DAP_, ∙10^4^) in mixtures of water and DES containing choline chloride and ethylene glycol (ETG) at various temperatures. In the first column, x*_ETG_ denotes the mole fraction of DES in solute-free mixtures with water. Standard deviation values are given in parentheses. Table S1.5. Mole fraction solubility of dapsone (x_DAP_, ∙10^4^) in mixtures of water and DES containing choline chloride and diethylene glycol (DEG) at various temperatures. In the first column, x*_DEG_ denotes the mole fraction of DES in solute-free mixtures with water. Standard deviation values are given in parentheses. Table S1.6. Mole fraction solubility of dapsone (x_DAP_, ∙10^4^) in mixtures of water and DES containing choline chloride and triethylene glycol (TEG) at various temperatures. In the first column, x*_TEG_ denotes the mole fraction of DES in solute-free mixtures with water. Standard deviation values are given in parentheses. Section S2. The Orange data mining. S2.1. General Information. S2.2. Orange collection. File 1: Spreadsheet file with dapsone solubility data. File 2: Orange data mining software file with dataset and performed analysis.
