# Supplementary material for: Experimental and Theoretical Insights into the Intermolecular Interactions in Saturated Systems of Dapsone in Conventional and Deep Eutectic Solvents
Source: Molecules. 2024 Apr 11;29(8):1743. doi: 10.3390/molecules29081743 (PMC11051893; doi:10.3390/molecules29081743)
Supplement: Supplementary file 1 [file molecules-29-01743-s001.zip › DAP3_SI.pdf]

Supplementary Materials

S1. Collection of experimental data of dapsone solubility 2

Table S1.1. Mole fraction solubility of dapsone (x_{DAP}, $\cdot 10^4$) in mixtures of water and DES containing choline chloride and 1,3-butanediol (B3D) at various temperatures. In the first column, x^*_{B3D} denotes the mole fraction of DES in solute-free mixtures with water. Standard deviation values are given in parentheses. 2

Table S1.2. Mole fraction solubility of dapsone (x_{DAP}, $\cdot 10^4$) in mixtures of water and DES containing choline chloride and 1,2-propanediol (P2D) at various temperatures. In the first column, x^*_{P2D} denotes the mole fraction of DES in solute-free mixtures with water. Standard deviation values are given in parentheses. 2

Table S1.3. Mole fraction solubility of dapsone (x_{DAP}, $\cdot 10^4$) in mixtures of water and DES containing choline chloride and glycerol (GLY) at various temperatures. In the first column, x^*_{GLY} denotes the mole fraction of DES in solute-free mixtures with water. Standard deviation values are given in parentheses. 2

Table S1.4. Mole fraction solubility of dapsone (x_{DAP}, $\cdot 10^4$) in mixtures of water and DES containing choline chloride and ethylene glycol (ETG) at various temperatures. In the first column, x^*_{ETG} denotes the mole fraction of DES in solute-free mixtures with water. Standard deviation values are given in parentheses. 3

Table S1.5. Mole fraction solubility of dapsone (x_{DAP}, $\cdot 10^4$) in mixtures of water and DES containing choline chloride and diethylene glycol (DEG) at various temperatures. In the first column, x^*_{DEG} denotes the mole fraction of DES in solute-free mixtures with water. Standard deviation values are given in parentheses. 3

Table S1.6. Mole fraction solubility of dapsone (x_{DAP}, $\cdot 10^4$) in mixtures of water and DES containing choline chloride and triethylene glycol (TEG) at various temperatures. In the first column, x^*_{TEG} denotes the mole fraction of DES in solute-free mixtures with water. Standard deviation values are given in parentheses. 3

S2. The Orange data mining 4

S2.1. General information 4

S2.2. Orange collection 4

S1. Collection of experimental data of dapsone solubility

Table S1.1. Mole fraction solubility of dapsone (x_{DAP}, $\cdot 10^4$) in mixtures of water and DES containing choline chloride and 1,3-butanediol (B3D) at various temperatures. In the first column, x^*_{B3D} denotes the mole fraction of DES in solute-free mixtures with water. Standard deviation values are given in parentheses.

x^*_{B3D}	dapsone solubility (x_{DAP}, $\cdot 10^4$)			
	**298.15 K**	**303.15 K**	**308.15 K**	**313.15 K**
**0.0**	0.03 (<±0.01)	0.04 (<±0.01)	0.05 (<±0.01)	0.06 (<±0.01)
**0.1**	62.37 (±1.04)	79.84 (±0.55)	109.79 (±0.95)	139.84 (±1.16)
**0.2**	122.17 (±0.89)	161.80 (±1.02)	209.42 (±4.98)	267.96 (±4.68)
**0.3**	181.50 (±2.47)	246.25 (±2.45)	321.56 (±5.44)	420.65 (±8.68)
**0.4**	266.78 (±1.83)	359.99 (±7.20)	472.67 (±3.45)	604.06 (±7.33)
**0.5**	371.55 (±2.68)	495.96 (±4.59)	660.95 (±5.33)	846.50 (±4.85)
**0.6**	504.83 (±9.66)	669.01 (±6.01)	884.13 (±8.04)	1158.39 (±8.10)
**0.7**	583.42 (±4.88)	782.94 (±6.83)	1015.31 (±7.56)	1311.68 (±14.89)
**0.8**	539.45 (±6.84)	724.92 (±10.49)	948.32 (±14.68)	1200.47 (±17.92)
**0.9**	507.58 (±12.94)	684.36 (±8.19)	877.05 (±15.62)	1123.00 (±14.16)
**1.0**	493.56 (±6.85)	662.31 (±10.99)	838.45 (±9.34)	1088.23 (±13.23)

Table S1.2. Mole fraction solubility of dapsone (x_{DAP}, $\cdot 10^4$) in mixtures of water and DES containing choline chloride and 1,2-propanediol (P2D) at various temperatures. In the first column, x^*_{P2D} denotes the mole fraction of DES in solute-free mixtures with water. Standard deviation values are given in parentheses.

x^*_{P2D}	dapsone solubility (x_{DAP}, $\cdot 10^4$)			
	**298.15 K**	**303.15 K**	**308.15 K**	**313.15 K**
**0.0**	0.03 (<±0.01)	0.04 (<±0.01)	0.05 (<±0.01)	0.06 (<±0.01)
**0.1**	53.83 (±0.58)	75.19 (±0.83)	96.16 (±0.59)	124.14 (±0.69)
**0.2**	108.22 (±0.87)	147.68 (±1.46)	195.88 (±2.81)	245.97 (±5.59)
**0.3**	164.54 (±1.66)	220.72 (±1.38)	285.36 (±2.42)	369.89 (±5.27)
**0.4**	237.33 (±0.93)	322.24 (±8.88)	418.69 (±4.20)	543.88 (±5.17)
**0.5**	333.61 (±5.75)	455.15 (±5.54)	587.98 (±5.30)	751.16 (±5.63)
**0.6**	449.20 (±8.52)	602.61 (±5.26)	780.98 (±2.49)	1026.59 (±9.74)
**0.7**	518.93 (±5.26)	698.60 (±4.94)	904.93 (±7.98)	1183.34 (±8.26)
**0.8**	505.19 (±5.78)	670.59 (±7.70)	875.38 (±10.41)	1131.49 (±5.14)
**0.9**	484.48 (±7.56)	642.31 (±7.06)	836.26 (±12.66)	1074.55 (±10.19)
**1.0**	469.06 (±13.84)	626.10 (±6.51)	804.31 (±10.3)	1028.47 (±6.01)

Table S1.3. Mole fraction solubility of dapsone (x_{DAP}, $\cdot 10^4$) in mixtures of water and DES containing choline chloride and glycerol (GLY) at various temperatures. In the first column, x^*_{GLY} denotes the mole fraction of DES in solute-free mixtures with water. Standard deviation values are given in parentheses.

x^*_{GLY}	dapsone solubility (x_{DAP}, $\cdot 10^4$)			
	**298.15 K**	**303.15 K**	**308.15 K**	**313.15 K**
**0.0**	0.03 (<±0.01)	0.04 (<±0.01)	0.05 (<±0.01)	0.06 (<±0.01)
**0.1**	79.53 (±0.84)	107.99 (±0.94)	142.39 (±1.02)	180.67 (±1.36)
**0.2**	164.04 (±1.33)	221.26 (±3.66)	292.65 (±2.55)	379.58 (±1.87)
**0.3**	290.11 (±2.90)	396.50 (±3.73)	516.89 (±5.51)	685.42 (±4.30)
**0.4**	430.36 (±4.45)	566.85 (±4.91)	764.66 (±6.21)	1013.44 (±5.16)
**0.5**	572.28 (±5.85)	769.90 (±3.61)	1013.58 (±13.28)	1350.94 (±17.79)
**0.6**	746.92 (±2.75)	1025.61 (±11.25)	1329.61 (±8.79)	1773.25 (±11.85)
**0.7**	804.63 (±9.11)	1100.25 (±10.32)	1455.17 (±13.47)	1899.83 (±16.85)
**0.8**	746.35 (±6.31)	1012.87 (±5.31)	1320.32 (±8.89)	1743.28 (±13.32)
**0.9**	700.92 (±5.84)	937.40 (±13.14)	1193.42 (±14.14)	1570.06 (±16.98)
**1.0**	651.01 (±15.03)	877.95 (±8.38)	1131.98 (±8.83)	1459.30 (±13.15)

Table S1.4. Mole fraction solubility of dapsone (x_{DAP}, $\cdot 10^4$) in mixtures of water and DES containing choline chloride and ethylene glycol (ETG) at various temperatures. In the first column, x^*_{ETG} denotes the mole fraction of DES in solute-free mixtures with water. Standard deviation values are given in parentheses.

X^*_{ETG}	dapsone solubility (x_{DAP}, $\cdot 10^4$)			
	**298.15 K**	**303.15 K**	**308.15 K**	**313.15 K**
**0.0**	0.03 (<±0.01)	0.04 (<±0.01)	0.05 (<±0.01)	0.06 (<±0.01)
**0.1**	109.53 (±0.76)	151.44 (±2.08)	194.27 (±2.23)	248.92 (±4.54)
**0.2**	226.42 (±2.95)	306.99 (±4.05)	405.06 (±3.02)	522.33 (±5.07)
**0.3**	330.54 (±2.63)	449.67 (±5.17)	594.64 (±4.09)	790.26 (±6.90)
**0.4**	433.93 (±2.61)	588.64 (±4.94)	776.44 (±3.91)	1039.00 (±11.36)
**0.5**	553.36 (±3.66)	755.84 (±5.88)	1001.46 (±7.95)	1358.75 (±11.26)
**0.6**	682.14 (±6.58)	931.10 (±5.22)	1234.44 (±8.82)	1672.59 (±11.96)
**0.7**	749.75 (±6.13)	1039.30 (±7.33)	1388.49 (±10.87)	1851.74 (±15.61)
**0.8**	772.55 (±10.22)	1063.16 (±12.41)	1404.27 (±13.42)	1870.64 (±19.99)
**0.9**	735.78 (±5.99)	987.52 (±13.10)	1304.17 (±21.73)	1755.85 (±17.77)
**1.0**	709.62 (±7.48)	962.35 (±10.58)	1268.84 (±18.45)	1630.24 (±28.82)

Table S1.5. Mole fraction solubility of dapsone (x_{DAP}, $\cdot 10^4$) in mixtures of water and DES containing choline chloride and diethylene glycol (DEG) at various temperatures. In the first column, x^*_{DEG} denotes the mole fraction of DES in solute-free mixtures with water. Standard deviation values are given in parentheses.

X^*_{DEG}	dapsone solubility (x_{DAP}, $\cdot 10^4$)			
	**298.15 K**	**303.15 K**	**308.15 K**	**313.15 K**
**0.0**	0.03 (<±0.01)	0.04 (<±0.01)	0.05 (<±0.01)	0.06 (<±0.01)
**0.1**	142.79 (±1.34)	196.47 (±1.31)	240.47 (±6.24)	341.23 (±3.72)
**0.2**	293.64 (±2.32)	402.27 (±3.12)	530.50 (±20.21)	710.18 (±10.16)
**0.3**	429.91 (±6.25)	573.65 (±5.61)	750.27 (±27.45)	1014.16 (±8.79)
**0.4**	567.16 (±5.30)	758.70 (±8.99)	1009.33 (±35.22)	1329.12 (±10.98)
**0.5**	713.37 (±7.38)	977.85 (±8.46)	1307.93 (±61.39)	1761.01 (±19.34)
**0.6**	861.27 (±7.35)	1174.28 (±10.97)	1556.20 (±66.95)	2184.80 (±21.51)
**0.7**	1011.52 (±9.93)	1362.79 (±11.24)	1823.10 (±61.70)	2442.78 (±17.23)
**0.8**	1046.86 (±6.77)	1398.30 (±12.75)	1854.50 (±87.83)	2486.45 (±23.15)
**0.9**	1001.60 (±8.70	1341.92 (±13.82)	1794.23 (±59.12)	2342.22 (±28.82)
**1.0**	964.77 (±10.66)	1304.33 (±17.18)	1715.07 (±71.61)	2251.29 (±8.92)

Table S1.6. Mole fraction solubility of dapsone (x_{DAP}, $\cdot 10^4$) in mixtures of water and DES containing choline chloride and triethylene glycol (TEG) at various temperatures. In the first column, x^*_{TEG} denotes the mole fraction of DES in solute-free mixtures with water. Standard deviation values are given in parentheses.

X^*_{TEG}	dapsone solubility (x_{DAP}, $\cdot 10^4$)			
	**298.15 K**	**303.15 K**	**308.15 K**	**313.15 K**
**0.0**	0.03 (<±0.01)	0.04 (<±0.01)	0.05 (<±0.01)	0.06 (<±0.01)
**0.1**	172.50 (±2.55)	221.67 (±2.64)	287.27 (±2.58)	380.37 (±3.25)
**0.2**	338.51 (±3.86)	459.63 (±3.37)	608.42 (±7.15)	805.85 (±8.35)
**0.3**	493.36 (±3.27)	668.57 (±0.52)	886.18 (±11.29)	1179.22 (±11.79)
**0.4**	638.58 (±10.62)	867.03 (±5.26)	1138.95 (±11.64)	1510.80 (±14.21)
**0.5**	805.21 (±8.55)	1113.59 (±8.14)	1468.38 (±23.12)	1959.84 (±11.53)
**0.6**	996.75 (±7.31)	1302.02 (±5.69)	1745.87 (±13.08)	2357.64 (±18.37)
**0.7**	1177.70 (±8.39)	1587.70 (±8.14)	2119.30 (±21.21)	2745.81 (±18.66)
**0.8**	1209.58 (±10.2)	1625.49 (±7.35)	2153.65 (±7.86)	2782.89 (±15.83)
**0.9**	1132.08 (±7.89)	1507.01 (±13.43)	1993.30 (±18.76)	2591.67 (±12.56)
**1.0**	1075.01 (±12.51)	1442.15 (±12.52)	1887.38 (±26.36)	2460.21 (±12.43)

S2. The Orange data mining

S2.1. General information

The Orange data mining system is available free of charge at https://orangedatamining.com/. It is a unique blend of tools that is useful for both beginners and expert data scientists. The visual programming interface is designed for non-programmers to perform real data mining and some machine learning projects without any knowledge of coding. On the other hand, its architecture is built on top of Python libraries such as sklearn, enabling in-depth study and sophisticated model formulation for scientific purposes. Orange offers a component-based data mining approach that uses stacked widgets as fundamental building blocks to define workflows. The variety of components with predefined internal structures enables intuitive data retrieval, pre-processing, visualization, and modelling or evaluation of project results. Combining diverse widgets in a workflow allows for flexible and comprehensive data analysis from scratch. This section documents the applicability of inferring information based on dapsone solubility in various solvents. The authors encourage readers to download and install the package, which is straightforward and does not require additional instructions. After this preparation, double-clicking on the attached 'ows' file provides access to the entire dataset and complete analysis. This file allows for the back-verification of the presented data and their interpretation, as well as the extension of the analysis with additional details not included in the main manuscript. The visualization in this manuscript was created using the simple structure presented in Scheme S2.1.

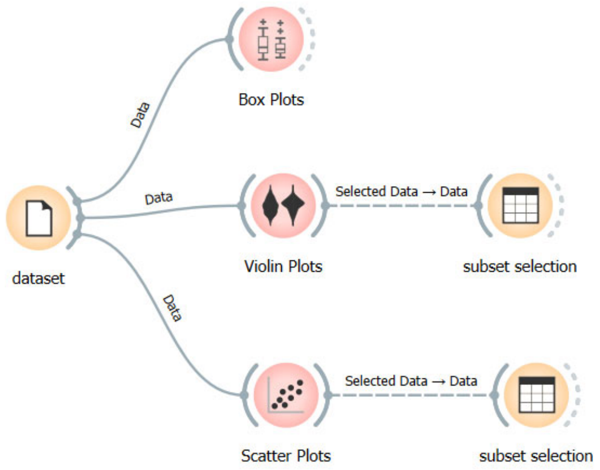

Scheme S2.1. Representation of the widget collection on the canvas of the Orange data mining file.

S2.2. Orange collection

All the data collected and used in this study are provided in two files. The first one is simply a spreadsheet with collected data on dapsone solubility in three studied solvent types, including new data measured for the purpose of this study. The second file comprises the same values in the format of Orange software. Double-clicking allows to directly access all collected numbers, including experimental solubility and computed affinity values. The basic tree widgets offer visualization of the data in the format presented in the main text. Additionally, there are interactive relationships between violin or scatter plots and datasets collecting only such records from the whole datasets, which are marked in the figure. This is very useful for quickly finding the systems fulfilling the marked condition. Since in the dataset a column with a description of the system is provided, it is very easy to

perform a variety of searches. For example, if one wants to inspect which solvents are characterized by the highest solvent-solvent affinities and the highest entropic contribution, one can mark them using a pointer as presented below:

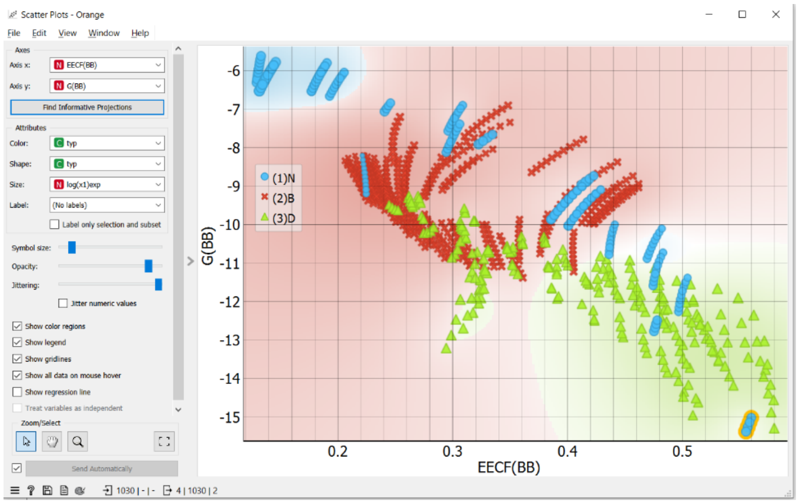

After this step, double-clicking on the marked subset selection

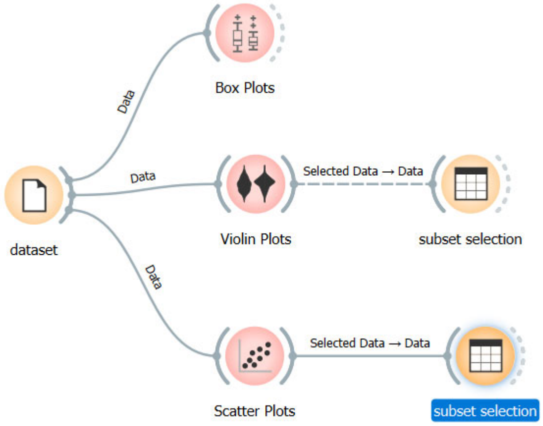

opens the window showing which data were marked on the scatter plot.
